# PACAP-38 and PACAP(6–38) Degranulate Rat Meningeal Mast Cells *via* the Orphan MrgB_3_-Receptor

**DOI:** 10.3389/fncel.2019.00114

**Published:** 2019-03-28

**Authors:** Sara Hougaard Pedersen, Sanne Hage la Cour, Kirstine Calloe, Frank Hauser, Jes Olesen, Dan Arne Klaerke, Inger Jansen-Olesen

**Affiliations:** ^1^Glostrup Research Institute, Danish Headache Center, Department of Neurology, Rigshospitalet Glostrup, Copenhagen, Denmark; ^2^Faculty of Health and Medical Sciences, University of Copenhagen, Copenhagen, Denmark; ^3^Department of Veterinary and Animal Sciences, Faculty of Health and Medical Sciences, University of Copenhagen, Frederiksberg C, Denmark; ^4^Cell and Neurobiology, Department of Biology, Faculty of Science, University of Copenhagen, Copenhagen, Denmark

**Keywords:** migraine, pituitary adenylate cyclase activating peptide, dura mater, *Xenopus laevis* oocytes, mast cell, Mas-related G-protein coupled receptor member B3, PAC1-receptor, two-electrode voltage clamp

## Abstract

Infusion of pituitary adenylate cyclase activating peptide-38 (PACAP-38) provokes migraine attacks in migraineurs and headache in non-migraineurs. Adverse events like long-lasting flushing and heat sensation can be terminated with oral antihistamine treatment, indicating the involvement of mast cell activation after PACAP-infusion. Degranulation of rat peritoneal mast cells was provoked by several isoforms of PACAP *via* previously unknown receptor pharmacology. The effect might thus be mediated either *via* specific splice variants of the PAC_1_-receptor or *via* an unknown receptor for PACAP-38. In the present study, we characterize degranulation of rat meningeal mast cells in response to PACAP-receptor ligands. Furthermore, we investigate if PACAP-38-induced mast cell degranulation is mediated *via* PAC_1_-receptor splice variants and/or *via* the orphan Mas-related G-protein coupled member B3 (MrgB_3_)-receptor. To address this, the pharmacological effect of different PACAP isoforms on meningeal mast cell degranulation was investigated in the hemisected skull model after toluidine blue staining followed by microscopic quantification. Presence of mRNA encoding PAC_1_-receptor splice variants and the MrgB_3_-receptor in rat mast cells was investigated by Reverse Transcriptase-Polymerase Chain Reaction (RT-PCR) analysis. The effect of PACAP isoforms on PAC_1_- and MrgB_3_-receptor-expressing *Xenopus laevis* oocytes were performed by two-electrode voltage-clamp (TEVC) electrophysiology. PACAP-38 is a more potent mast cell degranulating agent than Pituitary Adenylate Cyclase Activating Peptide-27 (PACAP-27) in the meninges. Presence of mRNA encoding the PAC_1_-receptor and its different splice variants could not be detected in peritoneal mast cells by RT-PCR, whereas the orphan MrgB_3_-receptor, recently suggested to be a mediator of basic secretagogues-induced mast cell degranulation, was widely present. In PAC_1_-receptor-expressing *Xenopus laevis* oocytes both PACAP-38, PACAP-27 and the specific PAC_1_-receptor agonist maxadilan were equipotent, however, only PACAP-38 showed a significant degranulatory effect on mast cells. We confirmed Pituitary Adenylate Cyclase Activating Peptide(6–38) [PACAP(6–38)] to be a PAC_1_-receptor antagonist, and we demonstrated that it is a potent mast cell degranulator and have an agonistic effect on MrgB_3_-receptors expressed in oocytes. The present study provides evidence that PACAP-induced mast cell degranulation in rat is mediated through a putative new PACAP-receptor with the order of potency being: PACAP-38 = PACAP(6–38) > > PACAP-27 = maxadilan. The results suggest that the observed responses are mediated *via* the orphan MrgB_3_-receptor.

## Introduction

Pituitary adenylate cyclase-activating peptide-38 (PACAP-38) is a 38-amino acid neuropeptide located in both sensory and parasympathetic perivascular nerve fibers (Moller et al., 1993; Mulder et al., 1994). A C-terminal truncated 27-amino acid (PACAP-27) version is endogenously present as well but is less abundant (Miyata et al., 1990; Arimura et al., 1991; Ogi et al., 1993). A 20-min intravenous infusion of PACAP-38 provokes migraine attacks in migraine patients as well as headache in non-migraineurs (Schytz et al., 2009). At present, three PACAP-receptors have been identified: PAC_1_, VPAC_1_ and VPAC_2_. The neurotransmitter vasoactive intestinal peptide (VIP) shares high amino acid sequence homology with PACAP and its affinity to VPAC_1_ and VPAC_2_ equals that of PACAP (Spengler et al., 1993; Pantaloni et al., 1996) whereas binding to the PAC_1_-receptor is 1,000 times lower (Miyata et al., 1989, 1990; Harmar et al., 1998). Interestingly, VIP only induces a mild headache and no migraine-like attacks in migraineurs (Rahmann et al., 2008), which leads to the suggestion that PACAP and the PAC_1_-receptor are key targets for future migraine treatment.

Infusion of PACAP-38 caused not only migraine attacks but also heat sensation and long-lasting flushing (Schytz et al., 2009). This is in line with PACAP-38 being a mast cell degranulator and mast cells have been suggested to play a role in migraine pathogenesis (Moskowitz, 1993; Levy et al., 2006, 2007). Degranulation of mast cells can be induced either by an allergen-IgE-dependent mechanism or *via* an IgE-independent mechanism. The latter mechanism can be activated by a group of molecules known as basic secretagogues. These molecules only share one physicochemical nature, their cationic property (Ferry et al., 2002). Several of these molecules are endogenous peptides and high concentrations are required for initiation of mast cell degranulation, an effect that involves pertussis toxin-sensitive G-proteins coupled to phospholipase C (PLC) activation (Ferry et al., 2002).

Inspired by clinical findings, we have previously characterized the degranulating effect of various PACAP-analogues on isolated rat peritoneal mast cells. Based on the expectation that degranulation is mediated through the PAC_1_-receptor, we found an unpredicted order of potency (Baun et al., 2012). In peritoneal mast cells, the PAC_1_-receptor antagonist Pituitary Adenylate Cyclase Activating Peptide(6–38) [PACAP(6–38)] caused mast cell degranulation that was as potent as PACAP-38 (Robberecht et al., 1992; Baun et al., 2012). Furthermore, the PAC_1_-receptor agonist maxadilan was ineffective (Baun et al., 2012).

Several PAC_1_-receptor splice variants have been cloned and characterized by ligand binding and signal transduction (Spengler et al., 1993; Pantaloni et al., 1996; Pisegna and Wank, 1996; Dautzenberg et al., 1999; Lutz et al., 2006). In 2006, Tatemoto et al. (2006) found the Mas-related G-protein coupled receptor member X_2_ (MrgX_2_) to be present in human mast cells. Mast cell degranulation induced by basic secretagogues appeared in the same concentrations as responses found in MrgX_2_-expressing cells. The rat counterpart of MrgX_2_ was found to be the Mas-related G-protein coupled receptor member B_3_ (MrgB_3_; Tatemoto et al., 2006). In the present study, we hypothesized that PACAP mediated degranulation by rat peritoneal and dural mast cells were either caused by a splice variant of the PAC_1_-receptor or *via* MrgB_3_-receptors.

## Materials and Methods

### Animals

A total of 115 male Sprague-Dawley rats weighing 320–440 g (Taconic Europe, Ejby, Denmark) were used in this study. The rats were group-housed under a 12-h light/dark cycle and allowed *ad libitum* access to a standard rodent diet and water. All rats were euthanized by inhalation of a CO_2_/O_2_-mixture followed by CO_2_ asphyxiation. Experimental procedures were approved by the National Danish Animal Experiments Inspectorate (License number 2014-15-0201-00256) and carried out in accordance with Danish legislation.

### Mast Cell Degranulation in Hemi-skull Preparations

Mast cell degranulation was performed as previously described (Pedersen et al., 2015). In brief, skulls were cut mid-sagittal and the brain halves were removed, leaving the dura mater undisturbed. This was followed by immediate addition of either 350 μl 0.1, 1 or 10 μM PACAP-38 (custom synthesis by Caslo Laboratory ApS, Lyngby, Denmark) or vehicle (saline) in phosphate buffered saline (PBS). After 30 s incubation, the reactions were terminated, and the skulls were fixated in 4% paraformaldehyde in phosphate buffered saline (PBS; Glostrup Hospital Pharmacy, Denmark). The dura mater was dissected from the skull, whole mounted on slides, and mast cells were visualized by staining with 0.1% acidified toluidine blue (Sigma Aldrich, Germany). Tissues were dehydrated in graded alcohols prior to cover slip mounting. The level of mast cell degranulation was evaluated by 400× magnification (Nikon Eclipse Ni microscope) by a researcher blinded to the treatment and was counted in 10 consecutive fields along the stem part of the middle meningeal artery. Mast cells were considered degranulated if an extensive dispersion of more than 10 extruded granules were localized outside the cell or if an extensive loss of staining gave the cell a “ghostly” look.

### Peritoneal Mast Cell Isolation and RNA-Extraction

Peritoneal mast cells were harvested from three rats by injecting 20 ml oxygenated buffer (137 mM NaCl, 2.7 mM KCl, 1 mM MgCl_2_, 0.5 nM CaCl_2_, 0.4 mM NaH_2_PO_4_, 10 mM HEPES and 5.6 mM glucose, pH 7.6) in the peritoneal cavity of asphyxiated rats. The cavity was then gently massaged and subsequently opened by midline incision for the lavage to be removed by pipetting. Cells were washed three times by sedimentation at 13°C by a 7 min centrifugation at 400 *g*. The pellet was re-suspended in 5 ml oxygenated buffer and layered on top of a BSA-Percoll^®^ density gradient (81%) containing 162 μl 35% bovine serum albumin (BSA), 8.1 ml Percoll^®^ (GE Healthcare, Buckinghamsure, UK), 580 μl distilled water and 1.16 ml salt solution (1.54 M NaCl, 27 mM KCl, 3.8 mM CaCl_2_). Cell types were separated by centrifugation at 225 *g* for 25 min at 13°C. The density gradient was discarded, and the pellet was again washed three times. The purity of mast cells was determined by histological characterization of the percentwise mast cell fraction. Only samples with a purity >90% were used for further analysis. Peritoneal mast cell RNA was extracted using the Isolation of Small and Large RNA Kit (Macherey-Nagel, Germany) in combination with TRIzol^®^ (Qiagen) according to manufacturer’s recommendations.

### Reverse Transcriptase-Polymerase Chain Reaction

cDNA was synthesized from 500 ng peritoneal mast cell RNA using the iScript cDNA Synthesis kit (BioRad) according to instructions. PAC_1_-receptor splice variants were identified in peritoneal mast cells and spinal cord using HotStarTaq^®^ DNA polymerase (QIAGEN) with 10 μM primer. The MrgB_3_-receptor was only tested in mast cell RNA. Primers against the Adcyap1r1 gene (NM_001270582.1, encoding the PAC_1_-receptor), RGD1560730 (XM_006229262.3, encoding the MrgB_3_-receptor), and β-actin were designed in Primer3 (Broad Institute) and tested for specificity by BLAST alignment tool (NCBI) and ordered from DNA Technology, Aarhus, Denmark (Table 1). PAC_1_-primers were specifically designed to span the extracellular N-terminal (exon 3 to exon 8), exon 14 (known as “hip”) and exon 15 (known as “hop”). β-actin was included as a positive control. The amplification protocol was as follows: initial heat activation at 95°C for 15 min, followed by 45 cycles with denaturation at 95°C for 1 min, annealing at variable temperatures depends on the primer set (50°C for β-actin and N-terminal, 56°C for MrgB_3_ and 66°C for Hip + Hop) for 1 min, and extension at 72°C for 1 min; final extension at 72°C for 10 min. Reverse Transcriptase-Polymerase Chain Reaction (RT-PCR) products were visualized by agarose gel electrophoresis.

**Table 1:** Primers designed for detection of the housekeeping gene β-actin, the N-terminal part and the hip-hop variants of the PAC_1_-receptor and the MrgB_3_-receptor.

Primer	Forward (5′→3′)	Reverse (5′→3′)	Product size
β-actin	tca aca ccc cag cca tgt acg	cag gaa gga agg ctg gaa gag	422 bp
N-terminal	tct gac tgc atc ttc aag aag	acc gac agg tag taa taa tcc	392 bp
Hip + Hop	ctt gta cag aag ctg cag tcc cca gac atg	ccg gtg ctt gaa gtc cat agt gaa gta acg gtt cac ctt	471bp / 387 bp
MrgB_3_	ccc ctg gaa tgt tct ttt gtg tag	aca gtg aaa aat gca gga act tcg	259 bp

*Each set of forward and reverse primer was designed in Primer3 (Broad Institute) against the Adcyap1r1 gene (NM_001270582.1), RGD1560730 (XM_006229262.3) encoding the MrgB_3_-receptor, and β-actin and tested for specificity by BLAST alignment tool (NCBI). The sizes of the amplified sequences are shown as base pair (bp)*.

### *In vitro* Transcription

DNA of the coding region of *Rattus Norvegicus* Adcyap1r1 variant 5 (NM_001270582.1) encoding the null-splice variant (short N-terminal and neither exon 14 or 15) of the PAC_1_-receptor (Vector: EX-Rn10199-M03) was ordered from GeneCopoeia. DNA encoding the RGD1560730 gene (XM_006229262.3, GenScript), was cloned into the pXOOM vector as previously described (Jespersen et al., 2002). DNA was purified using Plasmid DNA Purification NucleoBond Xtra Midi-kit (Macherey-Nagel). The inserts were fully sequenced (MWG Operon) to confirm the expected sequence (data not shown). Extracted plasmids were linearized down-stream the poly(A) segment using the *XhoI* restriction enzyme (New England Biolabs, Ipswich, MA, USA). RNA was *in* *vitro* transcribed by synthetization from the T7 RNA polymerase promoter using the mMessenger mMachine kit (Ambion) according to manufacturer’s protocol. Messenger RNA was purified using the MEGAclear kit (Ambion). Transcribed RNA integrity was assessed by agarose gel electrophoresis.

### Expression in *Xenopus laevis* Oocytes and Two-Electrode Voltage Clamp

Stage V and VI defolliculated *Xenopus laevis* oocytes were purchased from EcoCyte Bioscience (Dortmund, Germany) and kept in Kulori medium (90 mM NaCl, 4 mM KCl, 1 mM MgCl_2_, 1 mM CaCl_2_, and 5 mM Hepes, pH 7.4). Oocytes were micro-injected with 50 nl mRNA solution containing 30 ng RNA per oocyte and incubated at 19°C. Currents were measured after 2–5 days using a conventional two-electrode-voltage clamp (TEVC). The oocytes were placed in a 200 μl chamber and continuously exposed to a flow of Kulori medium with or without ligands (1 ml/min) while impaled with both a current and a voltage electrode filled with 3 M KCl and connected to an Oocyte Clamp Amplifier [Warner Instruments Corp. (OC-725 B) and a PC-interface (Digidata1440A, Molecular Devices)]. Current amplitude in absence or presence of ligands were analyzed using pClamp 10.2 software (Molecular Devices). All experiments were performed with oocyte membrane voltages constantly clamped to −70 mV and the temperature was kept between 19 and 22°C. In activation experiments, non-responding as well as low-responding (<10 nA) oocytes were excluded from the dataset. Ligands PACAP-38, PACAP-27, PACAP(6–38) were custom synthetized by Caslo (Lyngby, Denmark) while maxadilan was purchased from Bachem (Bubendorf, Switzerland).

Due to receptor desensitization, it was not possible to repeat measurements on individual oocytes, so only one dose could be tested per egg. Different batches of oocytes showed different expression levels and all figures are based on several batches of oocytes.

### Statistical Analysis

Concentration-response curves for both oocytes and dural mast cells were analyzed for overall effects of PACAP-38 and -27 by two-way analyses of variance (ANOVA) followed by Sidak’s test for multiple pairwise comparisons. Effects of PAC_1_-receptor ligands on mast cell degranulation or on receptor-expressing oocytes were analyzed with a one-way ANOVA followed by Tukey’s multiple comparisons test. The effect of PACAP-38 and PACAP(6–38) on MrgB_3_-expressing oocytes were analyzed by an unpaired two-tailed *t*-test. Differences between groups were considered significant when *p* < 0.05 and data are presented as mean with standard error of the mean (±SEM). GraphPad Prism 7 (GraphPad Prism Software, San Diego, CA, USA) was used for statistical analysis.

## Results

### Effects of PACAP-38 and PACAP-27 on Dura Mast Cell Degranulation

PACAP-provoked mast cell degranulation was characterized by stimulating the dura mater with PACAP-38, PACAP-27 or saline in concentrations ranging from 0.1 to 10 μM (*n* = 5–6; Table 2). PACAP-38 stimulation resulted in an eight-fold increase and highly significant mast cell degranulation at the 10 μM concentration (*p* < 0.0001). However, even at the highest tested concentration, PACAP-27 did not induce mast cell degranulation that was significantly different from saline treatment.

**Table 2:** The number of degranulated meningeal mast cells in % of total number of mast cells after 30 s incubation with either vehicle, PACAP-38 or PACAP-27 in concentrations ranging from 0.1 to 10 μM.

Conc. (μM)	Number of exp.	Vehicle to PACAP-38 (% degranulation)	PACAP-38 (% degranulation)	Vehicle to PACAP-27 (% degranulation)	PACAP-27 (% degranulation)
0.1	5	8.5 ± 3.0	12.4 ± 3.8	4.7 ± 1.9	7.1 ± 3.2
1.0	6	7.2 ± 1.3	17.9 ± 3.7	7.2 ± 2.0	16.9 ± 5.5
10.0	6	6.2 ± 1.7	48.4 ± 6.2****	5.9 ± 1.3	12.1 ± 3.8

Values are presented as means ± SEM. Statistical analysis (Two-way ANOVA followed by Sidak’s test) was performed to find significant differences in mast cell degranulation induced by each substance in each concentration. Significance is given as ****p < 0.0001.

### Splice Variants of PAC_1_-Receptor in Rat Peritoneal Mast Cells

**Figure 1:**
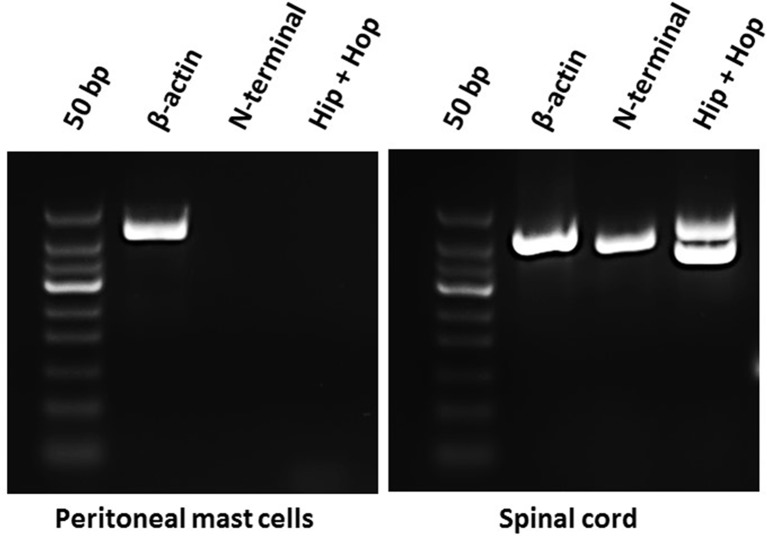
Agarose gel electrophoresis showing the absence of mRNA-expression of PAC_1_-receptor splice variants in rat peritoneal mast cells (left) and their presence in rat spinal cord (right) using primers directed towards the N-terminal part of the PAC_1_-receptor (392 bp) and exon 14 and 15 of the Hop-Hop variant of the PAC_1_-receptor (upper amplicon 471 bp and lower 387 bp). Primers detecting β-actin (422 bp) was used as a positive control and was present in both tissues. The experiment was performed in peritoneal mast cells from three rats.

Because mast cell degranulation induced by increasing concentrations of the different PACAP-isoforms did not follow the known order of potencies for PAC_1_-, VPAC_1_- or VPAC_2_-receptors, we investigated the presence of PAC_1_-receptor mRNA and possible splice variants by using RT-PCR (Figure 1). As a positive control, we included rat spinal cord tissue in which the PAC_1_-receptor previously has been localized (Dickinson et al., 1999). We performed RT-PCR analysis targeted to several areas involved in splice variation of the PAC_1_-receptor (N-terminal, exon 14 and exon 15) and found the PAC_1_-receptor to be absent in mast cells and present in spinal cord (Figure 1). The lack of PAC_1_-receptor mRNA expression in rat peritoneal mast cells indicates mast cell degranulation to be mediated *via* a non-PAC_1_-receptor.

### mRNA Expression of MrgB_3_-Receptor in Rat Peritoneal Mast Cells

**Figure 2:**
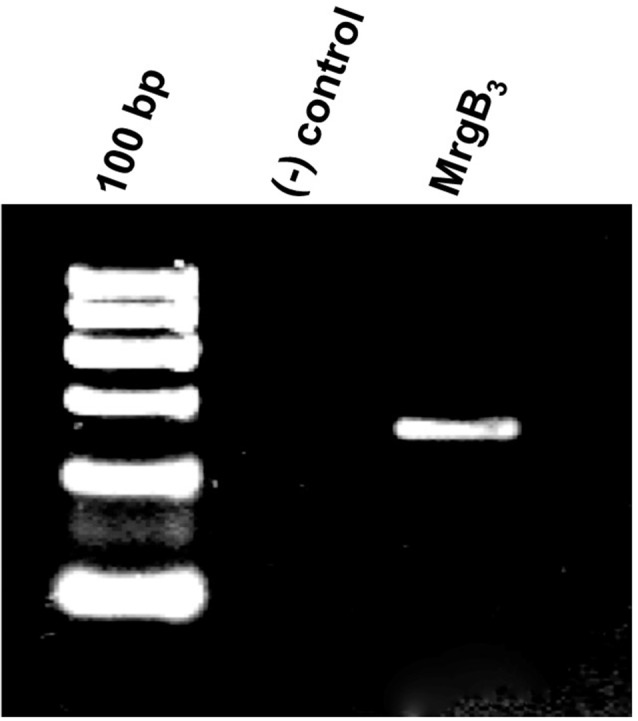
Agarose gel electrophoresis showing the RT-PCR-product corresponding the presence of mRNA encoding the MrgB_3_ receptor (259 bp) in rat peritoneal mast cells. No band is seen in the negative control [(−) control] where mRNA was not reverse transcribed to cDNA prior to amplification. The experiment was performed in peritoneal mast cells from three rats.

The PAC_1_-receptor was not expressed in rat peritoneal mast cells. Thus, we investigated a possible expression of the MrgB_3_-receptor as previously shown (Tatemoto et al., 2006). Using primers directed towards MrgB_3_-receptor mRNA, we found it to be expressed in rat peritoneal mast cells (Figure 2). Therefore, we decided to study the effect of PACAP isoforms on MrgB_3_-receptors and to compare the pharmacological profile on the PAC_1_-receptor using TEVC in the *Xenopus laevis* oocyte expression system.

### Effects of PACAP-38 and -27 on PAC_1_- and MrgB_3_-Receptors Expressed in *Xenopus laevis* Oocytes

**Figure 3:**
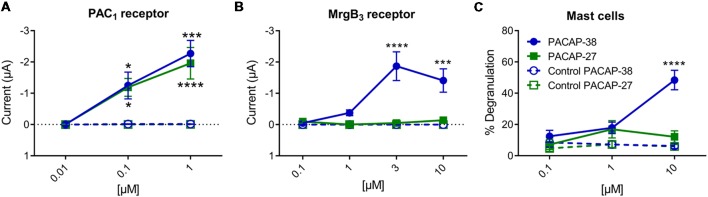
**(A)** Two-electrode voltage clamp performed on *Xenopus laevis* oocytes expressing the PAC_1_-receptor (*n* = 8–19, n_control_ = 6–16) showed that increasing concentrations (0.01–10 μM) of PACAP-38 and PACAP-27 cause a significant increase in membrane current. The inward currents are measured at a holding potential of −70 mV. In **(B)** the *Xenopus laevis* oocytes is expressing the MrgB_3_-receptor (*n* = 4–24, n_control_ = 3–13). In these oocytes only PACAP-38 induces a significant change in current (Vm = −70 mV). **(C)** Concentration-response curves of PACAP-38 and PACAP-27 on rat meningeal mast cell degranulation after 30 s stimulation at concentrations ranging from 0.1 to 10 μM. It was found that a significant mast cell degranulation only was observed after stimulation with PACAP-38. Values are given as percentage of degranulated mast cells of total number of counted mast cells (*n* = 5–6). *Represent *p* < 0.05, ***p < 0.001, and ****p < 0.0001 [two-way analysis of variance (ANOVA), Sidak’s multiple comparisons test]. All values are given as mean ± standard error of the mean (SEM).

Effects of PACAP-38 and PACAP-27 on PAC_1_- and MrgB_3_-receptors expressed in *Xenopus laevis* oocytes were investigated by TEVC. Upon addition of PACAP-38 and/or PACAP-27 at concentrations ranging from 0.01 to 1 μM to PAC_1_-receptor-expressing *Xenopus laevis* oocytes, a rapid concentration-depended inward current was observed (Figure 3A) consistent with activation of an endogenous Cl^−^ current following receptor activation. These findings confirmed the aforementioned studies of PACAP-38 and PACAP-27 being equipotent on the PAC_1_-receptor (Shivers et al., 1991; Pisegna and Wank, 1993). At 0.1 μM, PACAP-38 and PACAP-27-induced currents of −0.93 ± 3 μA (*n* = 19) and −1.04 ± 3 μA (*n* = 15), respectively. Stimulation with 1 μM of PACAP-38 and PACAP-27 resulted in currents of −2.27 ± 4 μA (*n* = 15) and −1.96 ± 5 μA (*n* = 13), respectively.

In MrgB_3_-receptor-expressing oocytes, PACAP-38, but not PACAP-27, induced a rapid concentration-depended inward current in the concentration range of 1–10 μM (*n* = 3–24). The maximum current induced by PACAP-38 was −1.87 ± 5 μA (*n* = 12) at 3 μM (Figure 3B). The maximum response to PACAP-27 was found at 10 μM resulting in a current of −0.14 ± 0.4 μA (*n* = 10), which was not significantly different from baseline. These rapid responses were not seen in un-injected oocytes. In concentrations between 0.1 and 3 μM no effect was observed in un-injected oocytes, indicating that *Xenopus laevis* oocytes do not endogenously express PAC_1_- or MrgB_3_-receptors. However, concentrations at 10 μM PACAP-38 (15 out of 19 oocytes) but not PACAP-27 occasionally caused a delayed long-lasting response in un-injected oocytes, which was distinct from the above described fast responses. Thus, 10 μM PACAP-38 was not included in the experiments. Taken together, expression of the PAC_1_-receptor and the MrgB_3_-receptor in *Xenopus laevis* oocytes shows that PAC_1_ is activated by PACAP-27 as well as PACAP-38, whereas the MrgB_3_-receptor is activated by PACAP-38 only. Thus, the activation profile found for MrgB_3_ resembles the effects of ligands observed for mast cell degranulation (Figure 3C). Taken together, mast cell degranulation induced by PACAP-38 and PACAP-27 in the meninges resembled mostly the current changes evoked in MrgB_3_-receptor transfected oocytes.

### Pharmacological Characterization of MrgB_3_- and PAC_1_-Receptor-Expressing Oocytes as Compared to Mast Cell Degranulation

#### Effect of PAC_1_-Receptor Agonist Maxadilan on MrgB_3_- and PAC_1_-Receptors

**Figure 4:**
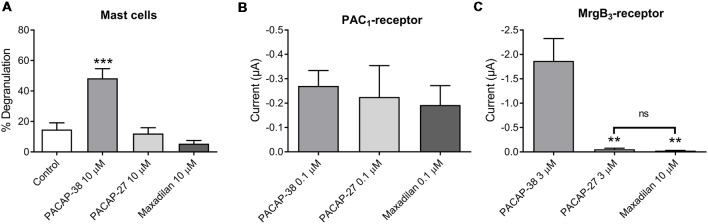
Effect of PACAP-38, PACAP-27 and maxadilan (PAC_1_-receptor agonist) on **(A)** rat meningeal mast cell degranulation following 30 s of 10 μM ligand stimulation. PACAP-38-induced a strong degranulation of meningeal mast cells. The mast cells were unresponsive to PACAP-27 and maxadilan. Values are given as percentage of degranulated mast cells from the total number of counted mast cells, *n* = 5–11. **(B)** Measurements on PAC_1_-receptor-expressing *Xenopus laevis* oocytes using two-electrode voltage clamp showed similar changes in current during 60 s perfusion of PACAP-38, PACAP-27 or maxadilan (all ligands 0.1 μM, *n* = 14–21). **(C)** In MrgB_3_-receptor-expressing oocytes PACAP-38 and PACAP-27 were perfused in a concentration of 3 μM and maxadilan at 10 μM (*n* = 8–12). Only PACAP-38 caused a change in current. All measurements were done at a holding potential of −70 mV. In **(A)** ***represent *p* < 0.001 as compared to control **(A)**. In **(C)**ns, *p* = 0.2879 (Mann-Whitney non-parametric *t*-test). **Represent *p* < 0.01 as compared to PACAP-38 (one-way ANOVA, Tukey’s multiple comparisons test). Values are given as mean ± SEM.

**Figure 5:**
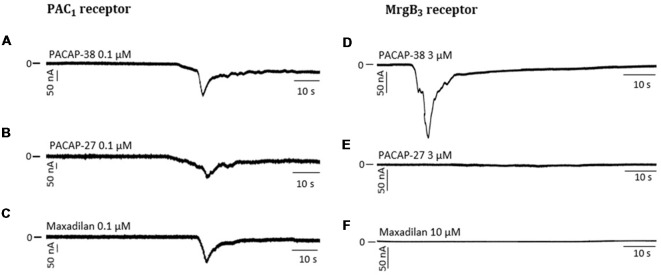
Representative traces showing a change in current after perfusion with PACAP-38 **(A)**, PACAP-27 **(B)** and maxadilan **(C)** on PAC_1_-receptor-expressing *Xenopus laevis* oocytes (**A** to **C**). In MrgB_3_-receptor-expressing *Xenopus laevis* oocytes (**D** to **F**) PACAP-38 caused a change in current **(D)** while there was no change after perfusion with PACAP-27 **(E)** and maxadilan **(F)**. The membrane potential was clamped to −70 mV. Scale bars represent 50 nA and 10 s.

Next, we characterized mast cell degranulation in response to the specific PAC_1_-receptor agonist maxadilan. We found that neither PACAP-27 nor maxadilan caused mast cell degranulation (12 ± 4%, *n* = 6 and 5 ± 2%, *n* = 5, respectively) despite the high concentration (10 μM) tested (Figure 4A). When we compared the potencies of the same agonists in a relevant concentration (0.1 μM) in PAC_1_-expressing *Xenopus laevis* oocytes, we observed that PACAP-27 and maxadilan induced changes in currents of −0.26 ± 1 μA (*n* = 14) and −0.21 ± 0.8 μA (*n* = 15), respectively. These responses were not significantly different (*p* = 0.8669) from currents observed after application of PACAP-38 (−0.27 ± 0.6 μA, *n* = 21; Figures 4B, 5A–C). In MrgB_3_-expressing oocytes, 3 μM PACAP-38 caused currents of −1.87 ± 5 μA (*n* = 12) that was significantly stronger than the effect induced by either 3 μM PACAP-27 (−0.06 ± 0.2 μA, *n* = 8) or up to 10 μM maxadilan (−0.03 ± 0 μA, *n* = 9; Figures 4C, 5D–F). Currents were not observed when PACAP-38, PACAP-27 or maxadilan were added to un-injected oocytes in the same concentrations (*data not shown*). Thus, this series of experiments conclude that maxadilan, PACAP-27 and PACAP-38 activates the PAC_1_-receptor with apparently equal potencies, whereas maxadilan does not activate MrgB_3_-receptors.

#### Effect of PAC_1_-Receptor Antagonist PACAP(6–38) on MrgB_3_- and PAC_1_-Receptors

**Figure 6:**
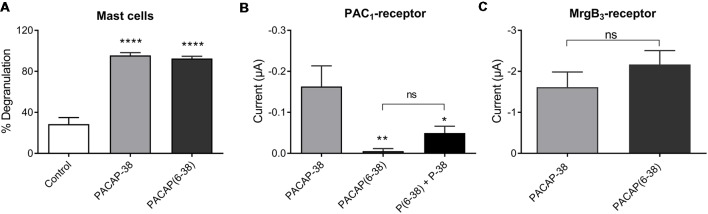
**(A)** The PAC_1_-receptor antagonist PACAP(6–38; 10 μM) is as potent as PACAP-38 (10 μM) to induce degranulation of rat meningeal mast cells. Values are given as percentage of degranulated mast cells (****p < 0.0001, one-way ANOVA followed by Tukey’s multiple comparisons test), *n* = 4–7. **(B)** PACAP(6–38; 0.1 μM) is unable to induce currents in PAC_1_-receptor-expressing *Xenopus laevis* oocytes using two-electrode voltage-clamp (TEVC) after 1 min perfusion of ligand. However, PACAP(6–38) caused a significant inhibition of PACAP-38 induced responses (*p < 0.05, **p < 0.01 one-way ANOVA followed by Tukey’s multiple comparisons test), *n* = 8–13. **(C)** In MrgB_3_-receptor-expressing *Xenopus laevis* oocytes PACAP(6–38; 3 μM) was equipotent to PACAP-38 (3 μM) to elicit currents (ns, *p* = 0.2944, unpaired *t*-test), *n* = 21–27. All measurements were done at a holding potential of −70 mV. All values are given as mean ± SEM.

Pharmacological characterization of PACAP-mediated mast cell degranulation was studied using the PAC_1_-receptor antagonist PACAP(6–38). As previously shown in peritoneal mast cells this antagonist showed agonistic properties in meningeal mast cells by inducing a significant (*p* < 0.0001) and almost complete degranulation (93 ± 2%, *n* = 7) when administered in a concentration of 10 μM (Figure 6A). This response was very similar to degranulation induced by PACAP-38 (96 ± 3%, *n* = 5). In PAC_1_-receptor-expressing oocytes PACAP(6–38) in a concentration of 0.1 μM had, as expected, no effect (−5 ± 6 nA, *n* = 9). However, the effect of PACAP-38 (−0.18 ± 0.5 μA, *n* = 11) was significantly antagonized when PACAP(6–38) was administered together with PACAP-38 in 0.1 μM (−0.05 ± 0.02 μA, *n* = 10; Figure 6B). In MrgB_3_-receptor-expressing oocytes, 3 μM PACAP(6–38) induced a change in the current of −2.17 ± 3 μA (*n* = 27), which was not significantly different from the response induced by PACAP-38 (−1.62 ± 4 μA, *n* = 21; Figure 6C).

## Discussion

PACAP-38, but not the related peptide VIP, induces migraine headache in migraineurs suggesting the specific PACAP-receptor, PAC_1_, as a potential target for migraine treatment (Rahmann et al., 2008; Schytz et al., 2009). Furthermore, all participants in the clinical provocation studies experienced long-lasting flushing, especially on the face and trunk, which could be terminated by antihistamine treatment, suggesting the involvement of mast cell degranulation (Schytz et al., 2009). In a previous series of experiments performed on rat peritoneal mast cells, we found that PACAP-38, but not PACAP-27 and VIP, caused degranulation. In addition, we showed that the selective PAC_1_-receptor agonist maxadilan had no effect on mast cell degranulation. It was also found that the selective PAC_1_-receptor antagonist, PACAP(6–38), induced a pronounced mast cell degranulation (Baun et al., 2012). Based on these observations, we suggested that the PACAP-provoked meningeal mast cell degranulation is mediated through another receptor than the PAC_1_-receptor (Baun et al., 2012). Our results suggest that PACAP-induced degranulation of rat peritoneal and meningeal mast cells is mediated *via* the orphan MrgB_3_-receptor.

### Pharmacology of PACAP on Mast Cell Degranulation

The weak degranulating effect of VIP and PACAP-27 compared to the strong degranulation effect of PACAP-38 in rat mast cells is inconsistent with the previously reported equipotent profiles of PACAP-38, PACAP-27, and VIP on VPAC_1_- and VPAC_2_-receptors (Harmar et al., 2012). In migraineurs, VIP does not provoke migraine headache, which suggests VPAC_1_- and VPAC_2_-receptors to be of minor importance in comparison to the PAC_1_-receptor (Rahmann et al., 2008). Furthermore, the PAC_1_-receptor antagonist PACAP(6–38) has not been shown to have an affinity to VPAC_1_- and VPAC_2_-receptors (Harmar et al., 2012). Taken together, this leads us to rule out the possible involvement of VPAC_1_- and VPAC_2_-receptors in PACAP-mediated mast cell degranulation.

### Expression of PAC_1_-Receptor Splice Variants

Several different splice variants of the PAC_1_-receptor have been identified in rats. Splice sites in the extracellular N-terminal domain and the third intracellular loop account for fine tuning of ligand affinity and signal transduction through adenylate cyclase or PLC activation (Deutsch and Sun, 1992; Spengler et al., 1993). The presence of a 21-amino acid domain in the extracellular N-terminal domain (PAC_1_-full, short) impairs PACAP-27 binding (Pantaloni et al., 1996). Hip and hop (exon 14 and 15, respectively) insertions into the third intracellular loop are suggested to modulate G-protein coupling and favor PACAP-38 induced PLC activation *via* Gq-proteins as compared to PACAP-27 (Spengler et al., 1993; Blechman and Levkowitz, 2013). This could explain the difference found in PACAP-38 and PACAP-27 provoked mast cell degranulation. We, therefore, designed primers directed towards the N-terminal part and towards exon 14 and 15. However, using the RT-PCR analysis we were unable to identify PAC_1_-receptor expression in the mast cell transcriptome. To further confirm the validity of the primers, we made parallel RT-PCR experiments on mRNA from spinal cord showing the presence of both the N-terminal part and the hip-hop variants of the PAC_1_-receptor. Based on these findings, we suggest PACAP-38-provoked mast cell degranulation to act *via* a target distinct from PAC_1_-receptors.

### PACAP as a Basic Secretagogue

Degranulation of mast cells induced *via* the IgE-independent pathway is mediated by a variety of compounds collectively designated as basic secretagogues. This is a mechanism highly conserved among mammals and birds, which appoints it to be ancient and fundamental (Halpern and Wood, 1950; Taneike et al., 1988). In general, basic secretagogues are positively charged, although hydrophobic structured compounds causing rapid mast cell degranulation [within ~10–20 s through PLC stimulation, which is sensitive to Gi-protein inhibition, e.g., pertussis toxin (Ferry et al., 2002; Tatemoto et al., 2006)]. PACAP-38 induced an almost total mast cell degranulation within the first 10–20 s after application. The degranulation was impaired by the PLC-inhibitor U-73122, whereas adenylyl cyclase inhibitor SQ22536 was ineffective (Baun et al., 2012), indicating PLC activation as the responsible transduction pathway for PACAP-induced mast cell degranulation.

**Figure 7:**
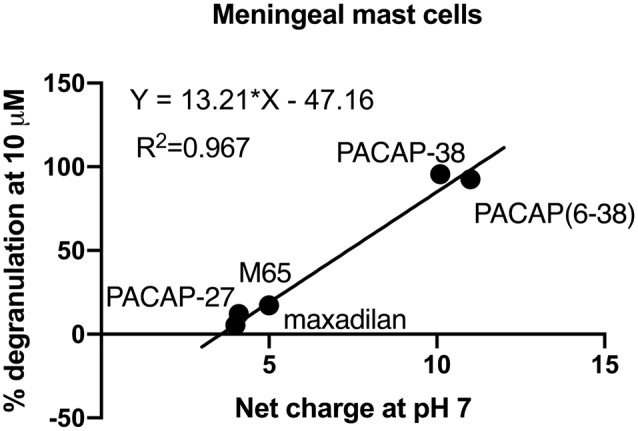
A correlation of 0.967 is obtained between the mean amount of degranulation induced by five PACAP isoforms (PACAP-38, PACAP-27, PACAP(6–38), maxadilan and M65), *n* = 5–11 in meningeal mast cells and their net charge at neutral pH. Calculations are performed using the Bachem Peptide Calculator (http://www.bachem.com/service-support/peptide-calculator/).

Interestingly, basic secretagogues seem to activate connective type mast cells independent of their putative receptor but only when applied in high concentrations (Ferry et al., 2002). Plotting the net charge of PACAP related molecules at neutral pH towards the level of degranulation induced by these PACAP analogues at 10 μM (Figure 7), we found a linear relationship with an R^2^ value close to 1. Thus, a high net charge of the molecules correlates with a high mast cell degranulating effect of the PACAP analogous tested, and several factors indicate that PACAP may act as a basic secretagogue to cause mast cell degranulation despite the absence of PAC_1_-receptors.

### Expression of MrgB_3_-Receptors

The exact mechanism of basic secretagogue-mediated mast cell degranulation remains unclear. The ability of basic secretagogues to act as direct activators of purified G-proteins as well as numerous failed attempts to identify an endogenously expressed receptor could suggest a receptor-independent mechanism of action (Mousli et al., 1990, 1994; Seebeck et al., 1998). However, this would require the ability of peptides, despite their positive charge, to diffuse across the membrane in order to reach intracellular G-proteins, and as this has not been shown, the hypothesis seems unlikely (Tatemoto et al., 2006). The identification of a basic secretagogue receptor has for a long time been sought but without success (Ferry et al., 2002). In 2006, Tatemoto et al. (2006) studied members of the Mrg family. These are G-protein coupled receptors and expressed in a subset of nociceptive sensory neurons, thus making them interesting targets (Dong et al., 2001; Lembo et al., 2002). Expression of MrgX_2_-receptors in humans and MrgB_3_-receptors in rats were shown to be present in connective type mast cells and with affinity to various peptides like PACAP(6–27), mast cell depleting peptide and [D-Trp^7,9,10^]-substance P could link these receptors to PACAP-mediated mast cell degranulation (Tatemoto et al., 2006). In a recent study, MrgX_2_-receptor (human) and MrgB_2_-receptor (which is the mouse orthologue of the human MrgX_2_-receptor and the rat MrgB_3_-receptor) was convincingly demonstrated to be mast cell-specific and responsible for inflammatory activation by basic secretagogues (McNeil et al., 2015). By RT-PCR, we found the MrgB_3_-receptor to be present in rat peritoneal mast cells and decided to study the effect of selected PACAP analogues on *Xenopus laevis* oocytes expressing MrgB_3_-receptors and to compare the responses to the effects obtained by the same PACAP analogues on PAC_1_-receptor-expressing oocytes.

### Experiments on MrgB_3_-Receptor and PAC_1_-Receptor-Expressing Oocytes as Compared to Mast Cell Degranulation

In rat MrgB_3_-receptor-expressing oocytes, we found that PACAP-38, but not PACAP-27 and maxadilan induces currents. The effect mediated by the different PACAP agonists in the oocytes had the same characteristics as those previously found to induce a significant degranulation of rat meningeal mast cells. The findings were also in line with our previous results from peritoneal mast cells (Baun et al., 2012). Importantly, the concentrations required for effects were in the 1–10 μM range. In PAC_1_-receptor-expressing oocytes, PACAP-38, PACAP-27 and maxadilan were equipotent and responses were found to be significant at 10 times lower concentrations.

In line with our results from peritoneal mast cells (Baun et al., 2012), we found the PAC_1_-receptor antagonist PACAP(6–38) both to be a potent degranulator of rat meningeal mast cells and to induce a significant current in MrgB_3_-receptor-expressing oocytes. Contrarily, PACAP(6–38) showed the predicted antagonistic effect on PACAP-38-induced currents in PAC_1_-receptor-expressing oocytes (Harmar et al., 2012). Taken together, our studies suggest mast cell degranulation to be mediated *via* MrgB_3_-receptors and not *via* the PAC_1_-receptor. However, the conclusion is limited by the fact that currently no selective antibodies or antagonists directed towards the MrgB_3_-receptor are available to provide the final pharmacological evidence.

Interestingly, a similar finding of PAC_1_-receptor pharmacology and functional observations in the rat trigemino-vascular system was reported. In these studies, PACAP-38, but neither VIP, PACAP-27 nor maxadilan, mediated the release of the sensory vasodilator peptide, calcitonin gene-related peptide (CGRP) from the trigeminal nucleus caudalis. Furthermore, the response to PACAP-38 seemed not to be mediated *via* PAC_1_-receptors due to lack of inhibition by the PAC_1_-receptor antagonist M65 (Jansen-Olesen et al., 2014). Future studies will have to rule out if MrgB_3_-receptors are involved in PACAP-38-induced CGRP release in trigeminal nucleus caudalis.

## Conclusion

In the present study, we found PACAP agonists and antagonists to have the same pharmacological effect in meningeal mast cells as previously found in peritoneal mast cells. By RT-PCR, we showed that there was no PAC_1_-receptor transcription in peritoneal mast cells thus excluding the possibility of the degranulating effect to be mediated *via* PAC_1_-receptor splice variants. However, we found mRNA encoding the rat MrgB_3_-receptor to be expressed in mast cells. This receptor was previously suggested to mediate mast cell degranulation in rat after application of basic secretagogues. This finding led us to investigate the effect of different PACAP analogues on *Xenopus laevis* oocytes expressing either PAC_1_- or MrgB_3_-receptors. The expressed MrgB_3_-receptor but not the PAC_1_-receptor share the same order of potency for PACAP analogues as found in rat peritoneal and meningeal mast cells. Thus, we hereby suggest the MrgB_3_-receptor to be a mediator for PACAP-induced mast cell degranulation.

## Data Availability

All datasets generated for this study are included in the manuscript.

## Author Contributions

IJ-O, SP and DK designed the research. KC and FH contributed with valuable intellectual input for improvement of the study. SP and SC performed the experiments. IJ-O, SP, CK, DK and SC performed data analysis. IJ-O and SP drafted the manuscript. JO, DK, KC, FH and SC read and corrected the manuscript. JO, IJ-O, SP and DK received financial support for the study. All authors read and approved the final manuscript.

## Conflict of Interest Statement

The authors declare that the research was conducted in the absence of any commercial or financial relationships that could be construed as a potential conflict of interest.

## Abbreviations

BSA: Bovine Serum Albumin
MrgB3: Mas-related G-protein coupled receptor member B3
MrgX2: Mas-related G-protein coupled receptor member X2
PACAP-27: Pituitary Adenylate Cyclase Activating Peptide-27
PACAP-38: Pituitary Adenylate Cyclase Activating Peptide-38
PACAP(6–38): Pituitary Adenylate Cyclase Activating Peptide(6–38)
PLC: Phospholipase C
RT-PCR: Reverse Transcriptase-Polymerase Chain Reaction
TEVC: Two-Electrode Voltage Clamp
VIP: Vasoactive Intestinal Peptide.

## References

[B1] Arimura A. Somogyvári-Vigh A. Miyata A. Mizuno K. Coy D. H. Kitada C. (1991). Tissue distribution of PACAP as determined by RIA: highly abundant in the rat brain and testes. Endocrinology 129, 2787–2789. 10.1210/endo-129-5-2787 1935809

[B2] Baun M. Pedersen M. H. Olesen J. Jansen-Olesen I. (2012). Dural mast cell degranulation is a putative mechanism for headache induced by PACAP-38. Cephalalgia 32, 337–345. 10.1177/0333102412439354 22421901

[B3] Blechman J. Levkowitz G. (2013). Alternative splicing of the pituitary adenylate cyclase-activating polypeptide receptor PAC1: mechanisms of fine tuning of brain activity. Front. Endocrinol. 4:55. 10.3389/fendo.2013.00055 23734144 PMC3659299

[B4] Dautzenberg F. M. Mevenkamp G. Wille S. Hauger R. L. (1999). N-terminal splice variants of the type I PACAP receptor: isolation, characterization and ligand binding/selectivity determinants. J. Neuroendocrinol. 11, 941–949. 10.1046/j.1365-2826.1999.00411.x 10583729

[B5] Deutsch P. J. Sun Y. (1992). The 38-amino acid form of pituitary adenylate cyclase-activating polypeptide stimulates dual signaling cascades in PC12 cells and promotes neurite outgrowth. J. Biol. Chem. 267, 5108–5113. 1312085

[B6] Dickinson T. Mitchell R. Robberecht P. Fleetwood-Walker S. M. (1999). The role of VIP/PACAP receptor subtypes in spinal somatosensory processing in rats with an experimental peripheral mononeuropathy. Neuropharmacology 38, 167–180. 10.1016/s0028-3908(98)00171-3 10193908

[B7] Dong X. Han S. Zylka M. J. Simon M. I. Anderson D. J. (2001). A diverse family of GPCRs expressed in specific subsets of nociceptive sensory neurons. Cell 106, 619–632. 10.1016/s0092-8674(01)00483-4 11551509

[B8] Ferry X. Brehin S. Kamel R. Landry Y. (2002). G protein-dependent activation of mast cell by peptides and basic secretagogues. Peptides 23, 1507–1515. 10.1016/s0196-9781(02)00090-6 12182955

[B9] Halpern B. N. Wood D. R. (1950). The action of promethazine (phenergan) in protecting mice against death due to histamine. Br. J. Pharmacol. Chemother. 5, 510–516. 10.1111/j.1476-5381.1950.tb00603.x 14801458 PMC1509989

[B10] Harmar A. J. Arimura A. Gozes I. Journot L. Laburthe M. Pisegna J. R.. (1998). International Union of Pharmacology. XVIII. Nomenclature of receptors for vasoactive intestinal peptide and pituitary adenylate cyclase-activating polypeptide. Pharmacol. Rev. 50, 265–270. 9647867 PMC6721840

[B11] Harmar A. J. Fahrenkrug J. Gozes I. Laburthe M. May V. Pisegna J. R.. (2012). Pharmacology and functions of receptors for vasoactive intestinal peptide and pituitary adenylate cyclase-activating polypeptide: IUPHAR review 1. Br. J. Pharmacol. 166, 4–17. 10.1111/j.1476-5381.2012.01871.x 22289055 PMC3415633

[B12] Jansen-Olesen I. Baun M. Amrutkar D. V. Ramachandran R. Christophersen D. V. Olesen J. (2014). PACAP-38 but not VIP induces release of CGRP from trigeminal nucleus caudalis via a receptor distinct from the PAC1 receptor. Neuropeptides 48, 53–64. 10.1016/j.npep.2014.01.004 24508136

[B13] Jespersen T. Grunnet M. Angelo K. Klaerke D. Olesen S.-P. (2002). Dual-function vector for protein expression in both mammalian cells and *Xenopus laevis* oocytes. Biotechniques 32, 536–540. 10.2144/02323st05 11911656

[B14] Lembo P. M. Grazzini E. Groblewski T. O’Donnell D. Roy M. O. Zhang J.. (2002). Proenkephalin A gene products activate a new family of sensory neuron—specific GPCRs. Nat. Neurosci. 5, 201–209. 10.1038/nn815 11850634

[B16] Levy D. Burstein R. Kainz V. Jakubowski M. Strassman A. M. (2007). Mast cell degranulation activates a pain pathway underlying migraine headache. Pain 130, 166–176. 10.1016/j.pain.2007.03.012 17459586 PMC2045157

[B15] Levy D. Burstein R. Strassman A. M. (2006). Mast cell involvement in the pathophysiology of migraine headache: a hypothesis. Headache 46, S13–S18. 10.1111/j.1526-4610.2006.00485.x 16927959

[B17] Lutz E. M. Ronaldson E. Shaw P. Johnson M. S. Holland P. J. Mitchell R. (2006). Characterization of novel splice variants of the PAC1 receptor in human neuroblastoma cells: consequences for signaling by VIP and PACAP. Mol. Cell. Neurosci. 31, 193–209. 10.1016/j.mcn.2005.09.008 16226889

[B18] McNeil B. D. Pundir P. Meeker S. Han L. Undem B. J. Kulka M.. (2015). Identification of a mast-cell-specific receptor crucial for pseudo-allergic drug reactions. Nature 519, 237–241. 10.1038/nature14022 25517090 PMC4359082

[B19] Miyata A. Arimura A. Dahl R. R. Minamino N. Uehara A. Jiang L.. (1989). Isolation of a novel 38 residue-hypothalamic polypeptide which stimulates adenylate cyclase in pituitary cells. Biochem. Biophys. Res. Commun. 164, 567–574. 10.1016/0006-291x(89)91757-9 2803320

[B20] Miyata A. Jiang L. Dahl R. D. Kitada C. Kubo K. Fujino M.. (1990). Isolation of a neuropeptide corresponding to the N-terminal 27 residues of the pituitary adenylate cyclase activating polypeptide with 38 residues (PACAP38). Biochem. Biophys. Res. Commun. 170, 643–648. 10.1016/0006-291x(90)92140-u 2383262

[B21] Moller K. Zhang Y. Z. Hakanson R. Luts A. Sjölund B. Uddman R.. (1993). Pituitary adenylate cyclase activating peptide is a sensory neuropeptide: immunocytochemical and immunochemical evidence. Neuroscience 57, 725–732. 10.1016/0306-4522(93)90018-b 7508577

[B22] Moskowitz M. A. (1993). Neurogenic inflammation in the pathophysiology and treatment of migraine. Neurology 43, S16–S20. 8389008

[B23] Mousli M. Bueb J. L. Bronner C. Rouot B. Landry Y. (1990). G protein activation: a receptor-independent mode of action for cationic amphiphilic neuropeptides and venom peptides. Trends Pharmacol. Sci. 11, 358–362. 10.1016/0165-6147(90)90179-c 2122563

[B24] Mousli M. Hugli T. E. Landry Y. Bronner C. (1994). Peptidergic pathway in human skin and rat peritoneal mast cell activation. Immunopharmacology 27, 1–11. 10.1016/0162-3109(94)90002-7 7515863

[B25] Mulder H. Uddman R. Moller K. Zhang Y. Z. Ekblad E. Alumets J.. (1994). Pituitary adenylate cyclase activating polypeptide expression in sensory neurons. Neuroscience 63, 307–312. 10.1016/0306-4522(94)90025-6 7898655

[B26] Ogi K. Miyamoto Y. Masuda Y. Habata Y. Hosoya M. Ohtaki T.. (1993). Molecular cloning and functional expression of a cDNA encoding a human pituitary adenylate cyclase activating polypeptide receptor. Biochem. Biophys. Res. Commun. 196, 1511–1521. 10.1006/bbrc.1993.2423 7902709

[B27] Pantaloni C. Brabet P. Bilanges B. Dumuis A. Houssami S. Spengler D.. (1996). Alternative splicing in the N-terminal extracellular domain of the pituitary adenylate cyclase-activating polypeptide (PACAP) receptor modulates receptor selectivity and relative potencies of PACAP-27 and PACAP-38 in phospholipase C activation. J. Biol. Chem. 271, 22146–22151. 10.1074/jbc.271.36.22146 8703026

[B28] Pedersen S. H. Ramachandran R. Amrutkar D. V. Petersen S. Olesen J. Jansen-Olesen I. (2015). Mechanisms of glyceryl trinitrate provoked mast cell degranulation. Cephalalgia 35, 1287–1297. 10.1177/0333102415574846 25724914

[B29] Pisegna J. R. Wank S. A. (1993). Molecular cloning and functional expression of the pituitary adenylate cyclase-activating polypeptide type I receptor. Proc. Natl. Acad. Sci. U S A 90, 6345–6349. 10.1073/pnas.90.13.6345 8392197 PMC46925

[B30] Pisegna J. R. Wank S. A. (1996). Cloning and characterization of the signal transduction of four splice variants of the human pituitary adenylate cyclase activating polypeptide receptor. Evidence for dual coupling to adenylate cyclase and phospholipase C. J. Biol. Chem. 271, 17267–17274. 10.1074/jbc.271.29.17267 8663363 PMC6721843

[B31] Rahmann A. Wienecke T. Hansen J. M. Fahrenkrug J. Olesen J. Ashina M. (2008). Vasoactive intestinal peptide causes marked cephalic vasodilation, but does not induce migraine. Cephalalgia 28, 226–236. 10.1111/j.1468-2982.2007.01497.x 18254893

[B32] Robberecht P. Gourlet P. De Neef P. Woussen-Colle M. C. Vandermeers-Piret M. C. Vandermeers A.. (1992). Structural requirements for the occupancy of pituitary adenylate-cyclase-activating-peptide (PACAP) receptors and adenylate cyclase activation in human neuroblastoma NB-OK-1 cell membranes. Discovery of PACAP(6–38) as a potent antagonist. Eur. J. Biochem. 207, 239–246. 10.1111/j.1432-1033.1992.tb17043.x 1321043

[B33] Schytz H. W. Birk S. Wienecke T. Kruuse C. Olesen J. Ashina M. (2009). PACAP38 induces migraine-like attacks in patients with migraine without aura. Brain 132, 16–25. 10.1093/brain/awn307 19052139

[B34] Seebeck J. Kruse M. L. Schmidt-Choudhury A. Schmidt W. E. (1998). Pituitary adenylate cyclase activating polypeptide induces degranulation of rat peritoneal mast cells via high-affinity PACAP receptor-independent activation of G proteins. Ann. N Y Acad. Sci. 865, 141–146. 10.1111/j.1749-6632.1998.tb11172.x 9928006

[B35] Shivers B. D. Gorcs T. J. Gottschall P. E. Arimura A. (1991). Two high affinity binding sites for pituitary adenylate cyclase-activating polypeptide have different tissue distributions. Endocrinology 128, 3055–3065. 10.1210/endo-128-6-3055 2036976

[B36] Spengler D. Waeber C. Pantaloni C. Holsboer F. Bockaert J. Seeburg P. H.. (1993). Differential signal transduction by five splice variants of the PACAP receptor. Nature 365, 170–175. 10.1038/365170a0 8396727

[B37] Taneike T. Miyazaki H. Oikawa S. Ohga A. (1988). Compound 48/80 elicits cholinergic contraction through histamine release in the chick oesophagus. Gen. Pharmacol. 19, 689–695. 10.1016/0306-3623(88)90130-9 2463953

[B38] Tatemoto K. Nozaki Y. Tsuda R. Konno S. Tomura K. Furuno M.. (2006). Immunoglobulin E-independent activation of mast cell is mediated by Mrg receptors. Biochem. Biophys. Res. Commun. 349, 1322–1328. 10.1016/j.bbrc.2006.08.177 16979137

